# CMR Clinical Practice Patterns Across Four U.S. Medical Centers from 2010-2014

**DOI:** 10.1186/1532-429X-18-S1-P311

**Published:** 2016-01-27

**Authors:** Dipan J Shah, Eric Y Yang, John Heitner, Afshin Farzaneh-Far, Faisal Nabi, Han W Kim, Alexander Ivanov, Igor Klem, Anna Lisa Crowley, Michele Parker, Robert Judd, Raymond Kim

**Affiliations:** 1 grid.63368.380000000404450041Houston Methodist DeBakey Heart & Vascular Center, Houston, TX USA; 2 grid.39382.33000000012160926XBaylor College of Medicine, Houston, TX USA; 3 New York Methodist, Brooklyn, NY USA; 4 grid.185648.60000000121750319University of Illinois at Chicago, Chicago, IL USA; 5 grid.189509.c0000000100241216Duke University Medical Center, Durham, NC USA

## Background

Accurate data on CMR practice patterns is a prerequisite for planning strategies to grow clinical volumes within existing CMR clinical services, increase the number of hospitals that offer these services, and improving reimbursement.

## Methods

Data analysis was performed on a cloud-based system that is currently receiving de-identified searchable data from electronically-signed clinical reports with full DICOM datasets for 23,275 consecutive CMR exams performed at four geographically diverse U.S. medical centers from Jan 1, 2010 through Dec 31, 2014. At the time of abstract submission, 8,242 datasets have been analyzed, and analysis of all 23,275 is expected by the end of 2015. All data fields were derived from CMR reports that had been electronically signed by board-certified physicians with Level 3 CMR training. We analyzed: 1) patient characteristics and clinical indications for CMR scans; 2) use and complication rates for contrast agents; 3) use and complication rates for stress testing; and 4) billing data based on CPT codes.

## Results

**Figure 1:**
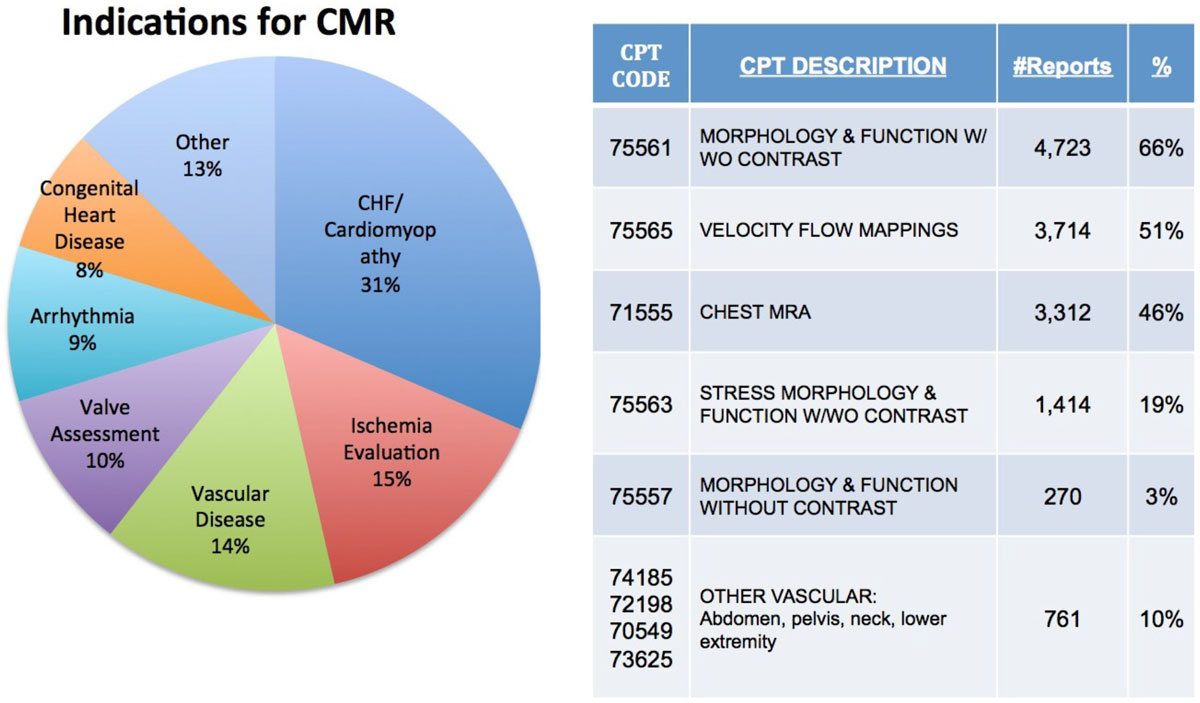
Figure 1

The median age of patients undergoing CMR was 59 years (IQR 20 years), 57% were male, 20% had a history of diabetes mellitus, 58% had a history of hypertension, 45% had a history of hyperlipidemia, and 9% were active smokers. Seventy-eight percent were outpatients and 22% were inpatients. The top reason for CMR scanning was CHF/cardiomyopathy, followed by ischemia evaluation, vascular disease, and valve assessment (Figure 1). Contrast agents were used in 84% of all scans. Contrast agent dose was: 0.1 mmol/kg (7.1%), 0.15 mmol/kg (69%) and 0.2 mmol/kg (24%). Stress testing was performed in 1,443 of the 8,242 patients (18%). The stress test agent was Regadenoson in 747 patients (51%) and adenosine in 696 (48%). Stress scans were terminated prematurely in 0.5% patients (n = 7) due to symptoms. No patient experienced a serious complication (death or MI) due to CMR stress testing. The top CPT code billed was 75561 (morphology with contrast, used in 66% of all scans), followed by 75565 (velocity flow, 51%) and 71555 (chest MRA, 46%). For patients with CPT code 71555 (chest MRA), the most common indication was aortic aneurysm/dissection or congenital heart disease. Commonly used CPT codes are noted in the figure, on average 2.0 CPT codes were billed per patient.

## Conclusions

CMR is clinically viable in the United States with the most common indications: heart failure/cardiomyopathy, ischemia evaluation, vascular disease, and valve assessment. CMR vasodilator stress testing appears remarkably safe in clinical practice.

